# Sexually Dimorphic and Intersex-Specific Transcriptional Responses in *Cherax quadricarinatus* Hepatopancreas Following Methyl Farnesoate Exposure

**DOI:** 10.3390/ijms27094005

**Published:** 2026-04-29

**Authors:** Jie Wei, Kunhao Hong, Yakun Wang, Zhuang Mai, Bai Liufu, Qiyao Su, Sikai Xu, Qiaoyan Zhou, Tianhui Jiao, Zikang Tu, Yayi Huang, Lingyun Yu

**Affiliations:** Key Laboratory of Tropical and Subtropical Fishery Resources Application and Cultivation, Ministry of Agriculture and Rural Affairs, Pearl River Fisheries Research Institute, Chinese Academy of Fishery Sciences, Guangzhou 510380, China; zjweijie@prfri.ac.cn (J.W.); m15775090244@163.com (K.H.); wykzkyky@163.com (Y.W.); mai9744@sina.com (Z.M.); cypressliufu@foxmail.com (B.L.); 15277124729@163.com (Q.S.); 2023710942@yangtzeu.edu.cn (S.X.); zhouqiaoyan@prfri.ac.cn (Q.Z.); jiaotianhui0730@163.com (T.J.); t453929526@outlook.com (Z.T.); q13362344580@163.com (Y.H.)

**Keywords:** *Cherax quadricarinatus*, methyl farnesoate, intersex, transcriptomic response

## Abstract

The redclaw crayfish (*Cherax quadricarinatus*) features a unique intersex phenotype—functional males harboring a female (ZW) genotype. This study investigates the sexually dimorphic transcriptomic responses of the hepatopancreas to acute methyl farnesoate exposure to decouple genotypic from phenotypic sex. We found that normal males prioritize enzymatic detoxification and steroidogenesis, whereas normal females prioritize energy conservation for reproductive preparation. Strikingly, intersex individuals exhibited a massive transcriptomic burst and paradoxical hormone receptor dynamics, exposing a fragile endocrine network driven by their inherent genotypic–phenotypic conflict. To survive severe MF-induced pharmacological stress, the intersex hepatopancreas actively suppresses lysosomal and apoptotic pathways, which we hypothesize serves as a compensatory mechanism to mitigate severe tissue damage. Instead, it deploys a compensatory architecture by hyperactivating amino acid biosynthesis, sulfur relay systems, and gap junctions to manage proteotoxic and oxidative stress. Co-expression network analysis identified *VCP*, *maf*, and *hdac8* as central regulatory hubs orchestrating this survival strategy through proteostasis, oxidative sensing, and epigenetic override. These findings suggest that the crustacean response to acute pharmacological challenge involves profound metabolic and epigenetic reprogramming, providing novel hypotheses for future functional studies.

## 1. Introduction

The redclaw crayfish (*Cherax quadricarinatus*) is a commercially and ecologically significant freshwater crustacean characterized by pronounced sexual dimorphism, with males typically exhibiting faster growth rates and larger body sizes than females [1,2]. Although it is fundamentally a gonochoristic species, *C. quadricarinatus* frequently exhibits a striking intersex phenomenon in natural populations, rendering it an exceptional biological model for elucidating crustacean sex determination and endocrine regulation [3]. Anatomically, intersex individuals uniquely possess both male and female gonopores and develop an ovotestis, which consists of functional testicular tissue accompanied by a vestigial ovary [4,5]. The most compelling aspect of this phenomenon is its underlying genetic paradox: while intersex crayfish function primarily and reproductively as males, rigorous chromosome-based sex-determination studies have confirmed that they possess a heterogametic female (WZ) genotype, in stark contrast to the homogametic (ZZ) genotype of normal males [3,6]. This profound genotypic–phenotypic incongruence creates a unique physiological tension that is strongly reflected at the molecular level. Recent joint transcriptomic and metabolomic analyses have demonstrated that intersex individuals present significant baseline deviations in steroid hormone synthesis, lipid metabolism, and the differential expression of critical sex-related genes, including key members of the cytochrome P450 family (e.g., *cyp3a40* and *cyp18a1*) [7]. Therefore, leveraging this intersex model offers an unprecedented opportunity to decouple genotypic sex from phenotypic sex, providing a critical framework for exploring sexually dimorphic molecular responses to exogenous endocrine challenges.

Methyl farnesoate (MF), a non-epoxidized sesquiterpenoid synthesized by the mandibular organ, serves as the crustacean equivalent of insect juvenile hormone and functions as a master endocrine regulator [8]. It exhibits profound pleiotropic effects throughout the crustacean life cycle, orchestrating critical physiological processes such as molting, morphogenesis, and metabolic homeostasis [9]. Notably, MF exerts a robust, yet sexually dimorphic, stimulatory effect on reproduction. In females, it acts as a potent gonadotropin, directly promoting ovarian maturation and vitellogenesis—a dynamic equally crucial across other decapod models like *Macrobrachium rosenbergii* [10]. Conversely, in males, elevated MF titers are intrinsically linked to the allometric growth of reproductive organs, the maintenance of distinct male morphotypes, and the stimulation of reproductive behaviors [11]. Beyond its independent reproductive functions, emerging evidence suggests that MF is deeply intertwined with sex determination and differentiation pathways. For instance, in branchiopods with environmental sex determination, MF acts as the primary switch programming embryos to develop into males [12]. Although male sexual differentiation in gonochoristic decapods like *C. quadricarinatus* is primarily driven by the insulin-like androgenic gland hormone (IAG) [13], the exogenous administration of a primary hormone like MF inevitably triggers a systemic endocrine perturbation. This perturbation likely forces a complex molecular crosstalk with the IAG signaling axis and other sex-related genetic networks [14]. Thus, utilizing MF as a targeted pharmacological probe provides a powerful tool to challenge the unique endocrine environment of intersex crayfish, probing how their inherent female (WZ) genotypic foundation reacts when a predominantly masculinized, IAG-driven physiological state is subjected to a profound hormonal surge.

To comprehensively capture the molecular ramifications of such endocrine disruptions, the target tissue must be meticulously selected; in crustaceans, the hepatopancreas is the premier candidate. Often oversimplified as a mere digestive gland, the hepatopancreas actually functions as the central metabolic and endocrine command hub, orchestrating energy partitioning, lipid mobilization, and systemic metabolic reprogramming [15,16]. Furthermore, it is intrinsically involved in the biosynthesis and biotransformation of steroid hormones, harboring an extensive repertoire of critical regulatory enzymes, including members of the cytochrome P450 (CYP) superfamily (such as CYP15A1 and CYP18A1) and hydroxysteroid dehydrogenases [17]. Most crucially, in the context of MF exposure, the hepatopancreas acts as the primary detoxification and hormone clearance center. It dictates the in vivo half-life of MF through the robust expression of specialized carboxylesterases—notably methyl farnesoate esterase (MFE) and juvenile hormone esterase-like (JHE-L) enzymes—which hydrolyze and neutralize excess circulating sesquiterpenoids [18]. Consequently, when the crayfish is challenged with an acute influx of exogenous MF, the hepatopancreas is forced into a state of intense transcriptomic activity to manage hormone detoxification, reallocate metabolic substrates, and mitigate cellular stress. Therefore, analyzing the hepatopancreatic transcriptome provides a profound molecular window into how different genotypic and phenotypic sexual backgrounds deploy distinct physiological and survival strategies under severe endocrine pressure.

At the molecular level, the genomic action of MF is mediated by an intracellular signaling cascade primarily driven by the basic helix-loop-helix Per-ARNT-Sim (bHLH-PAS) receptor Methoprene-tolerant (Met) and subsequent early-response transcription factors, such as Krüppel homolog 1 (Kr-h1) and Broad [19,20]. Crucially, recent transcriptomic advances have revealed that this hormone-receptor axis does not operate in a static cellular environment; rather, it is subjected to profound sexually dimorphic regulation. For instance, in the hepatopancreas of the *Litopenaeus vannamei*, an influx of MF elicits highly sex-biased transcriptomic responses starting at the receptor level, characterized by the sex-specific alternative splicing of *Met* and divergent expression trends of *Kr-h1*. More importantly, the downstream functional networks mobilized by MF diverge dramatically between sexes. The male hepatopancreatic transcriptome predominantly upregulates sugar and lipid metabolism alongside a massive induction of chitin metabolism genes (e.g., *CHIT1*), indicating a robust metabolic reprogramming geared toward structural remodeling and molting preparation. Conversely, the female response is characterized by the specific enrichment of miRNA biogenesis and immune-related pathways—such as the upregulation of *SMAD3* and *DDX17*—suggesting a prioritized strategy of anti-apoptosis, cellular protection, and reproductive energy conservation [21].

Despite the extensive documentation of MF’s independent roles in crustacean biology [8,9], and the recent elucidation of baseline transcriptomic and metabolomic differences between normal and intersex *C. quadricarinatus* [1,7], a profound knowledge gap remains. The scientific community currently lacks a comprehensive understanding of how hepatopancreatic metabolic reprogramming and endocrine detoxification networks are modulated by extreme sexual phenotypes under acute hormonal perturbation. Specifically, the dynamic transcriptomic response of the intersex model remains entirely unexplored. Because intersex redclaw crayfish possess a heterogametic female (WZ) genotype [3] yet operate within a highly masculinized physiological environment, their cellular machinery is subjected to a fundamental genotypic–phenotypic conflict. When challenged with an exogenous influx of a master hormone like MF, it is completely unknown how the intersex hepatopancreatic transcriptome will react: will it prioritize a male-biased metabolic response characterized by chitin and lipid mobilization, or will it unmask an underlying female-biased (WZ) immune and stress-regulatory network? Alternatively, it might exhibit a novel, chimeric compensatory pathway. Furthermore, since hormonal regulation is intrinsically dose-dependent, the specific transcriptomic thresholds that distinguish a normal physiological adaptation from a severe pharmacological stress response to varying MF concentrations remain uncharacterized in the family Parastacidae. Resolving these blind spots is imperative not only for advancing our evolutionary understanding of crustacean endocrine plasticity but also for discovering novel molecular targets for monosex aquaculture technologies.

Building upon these recognized gaps, the primary objective of this study is to elucidate the sexually dimorphic transcriptomic architecture of the *C. quadricarinatus* hepatopancreas following MF exposure, with a specific focus on the unique intersex phenotype. To isolate the fundamental hormonal response from the confounding metabolic background of active adult reproduction, juvenile crayfish were deliberately selected as the experimental model. Furthermore, rather than assessing environmental toxicity, we utilized MF strictly as an experimental probe. Guided by established pharmacological challenge protocols for decapod models [21], we employed a dual-dose MF injection strategy (1000 ng and 5000 ng) to precisely differentiate between a physiological adaptation, which mimics natural developmental hormonal surges, and a severe pharmacological stress response. A 24 h post-injection sampling window was established, representing an optimal kinetic timeframe to capture the robust secondary transcriptomic cascade without the signal being completely diluted by hepatopancreatic enzymatic clearance [22]. We hypothesize that the transcriptomic response to MF will be highly dose-dependent and strongly dictated by the underlying genotypic and phenotypic sexual framework. Specifically, we anticipate that while normal males and females will exhibit divergent metabolic and immune routing, the intersex individuals harboring a female (WZ) genotype within a masculinized physiological environment will unveil a novel, chimeric compensatory network. Under the extreme pharmacological pressure of the 5000-ng dose, these intersex individuals may even unmask a latent female-biased genetic stress response. Ultimately, these findings will provide unprecedented insights into the intersection of pharmacological endocrine challenge, metabolic reprogramming, and sexual dimorphism in decapod crustaceans.

## 2. Results

### 2.1. Overview of Transcriptome Data

To profile the hepatopancreatic transcriptome under MF exposure, 27 cDNA libraries were sequenced, encompassing normal females (F), normal males (M), and intersex individuals (J) across three MF doses (control [0 ng], 1000 ng, and 5000 ng) with three biological replicates. Sequencing yielded massive and consistent data, with raw reads ranging from 39,216,368 (J-CON-3) to 55,013,018 (F-1000-3). Stringent quality control retained a remarkable 99.99% of high-quality sequences (Clean Data) across all samples. Adapter contamination was strictly limited to 0.01%, while both low-quality sequences and unknown bases (N) approached 0.00%. This sequencing depth and purity establish a robust foundation for the precise quantification of low-abundance transcripts and the resolution of sex-specific expression heterogeneity (Table S1).

To assess biological variance and data reproducibility among the samples, Principal Component Analysis (PCA) was performed (Figure S1). The PCA plot demonstrated that the three biological replicates within each specific phenotype and treatment group, including the intersex control group (J-CON), clustered tightly together, indicating low intra-group variance and high experimental reliability. Notably, the intersex individuals exposed to MF (J-1000 and J-5000) exhibited a massive spatial shift away from their control baseline along the principal components compared to the normal male and female groups. This tight intra-group clustering combined with the profound inter-group separation confirms that the uniquely large number of differentially expressed genes (DEGs) observed in the intersex phenotype reflects a robust biological response to the pharmacological challenge, rather than a statistical artifact driven by baseline inter-individual noise.

### 2.2. Global Transcriptomic Overview and Massive Activation in Intersex Crayfish

**Figure 1:**
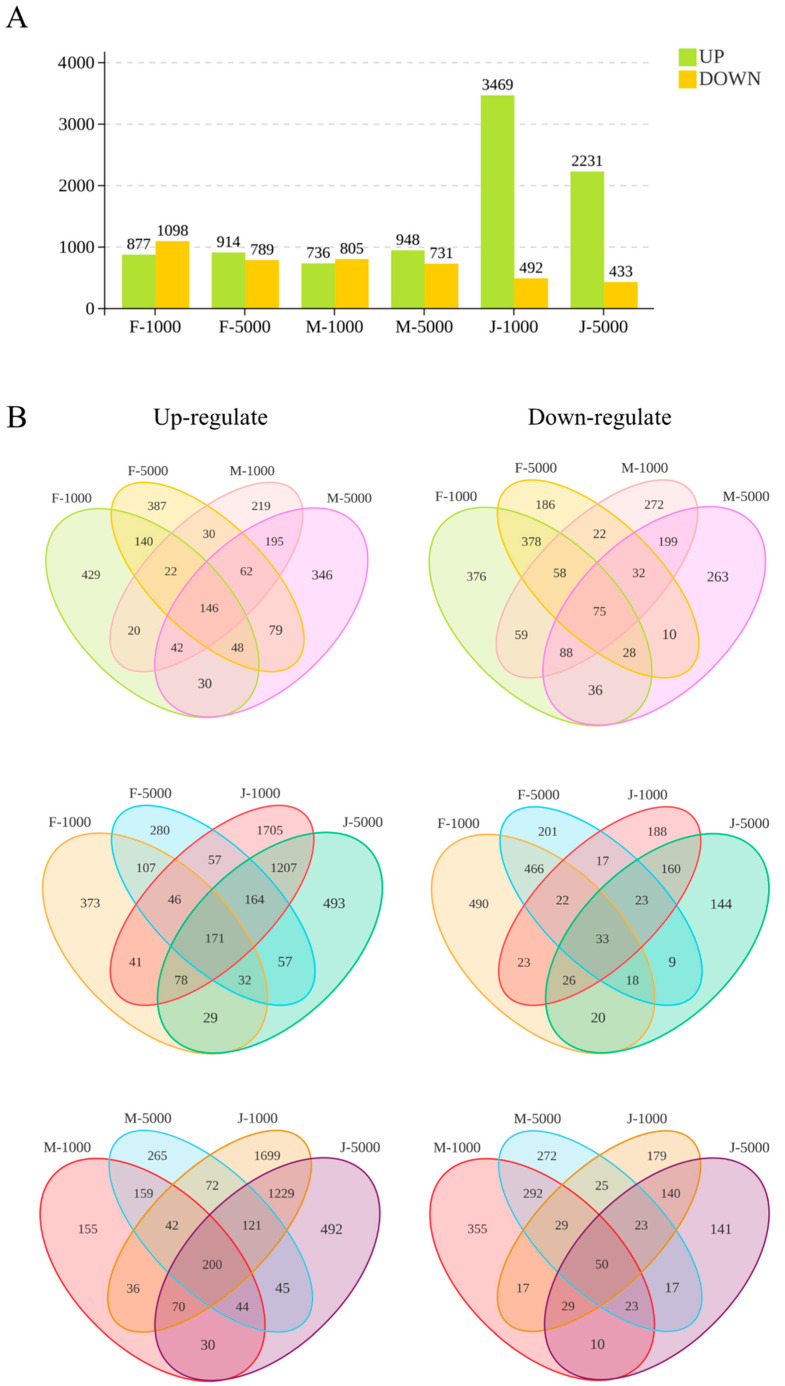
Statistical summary (**A**) and Venn diagrams (**B**) detailing the overlap of DEGs among different sex and MF dose combinations.

The results of overall gene expression changes after MF exposure showed that MF injection caused distinct transcriptomic responses across all groups. In normal females, there were 877 up-regulated and 1098 down-regulated genes at the 1000 ng dose (F-1000), and 914 up-regulated and 789 down-regulated genes at the 5000 ng dose (F-5000). In normal males, the 1000 ng dose (M-1000) resulted in 736 up-regulated and 805 down-regulated genes, while the 5000 ng dose (M-5000) led to 948 up-regulated and 731 down-regulated genes. Notably, the intersex crayfish exhibited a much stronger transcriptomic response. At the 1000 ng dose (J-1000), the number of up-regulated genes reached a massive peak of 3469, which was significantly higher than that in normal males and females, along with 492 down-regulated genes. Under the 5000 ng dose (J-5000), intersex individuals continued to show a strong activation, with 2231 up-regulated and 433 down-regulated genes (Figures 1 and S2A).

Additionally, a Venn diagram was generated to analyze the overlap of DEGs among different groups. The diagram revealed that a large number of DEGs were unique to each specific sex and dose combination, indicating that the molecular response to MF is highly specific to the sexual phenotype rather than a general stress response (Figure S2B).

### 2.3. Sexually Dimorphic Responses of Key Components in Hormone-Related Pathways

In-depth transcriptomic analysis revealed profound sexually dimorphic responses in core hormone signaling pathways under varying MF concentrations (Table S2). The primary MF receptor *Met* (ncbi_128693905) exhibited distinct basal levels: moderate in normal females (F-CON, 0.408–0.641 FPKM), lower in males (M-CON, 0.141–0.326 FPKM), and nearly silenced in intersex individuals (J-CON, 0.038–0.112 FPKM). Notably, high-dose MF (5000 ng) triggered an extreme compensatory rebound in the intersex group, peaking at 0.869 FPKM. The downstream primary response factor *Kr-h1* (ncbi_128701337) showed a hyperactive baseline in the intersex control (~425–437 FPKM), which was more than double that of normal females (~194–202 FPKM). Following exogenous MF exposure, female *Kr-h1* displayed a classic dose-dependent activation, peaking at ~383 FPKM, whereas intersex individuals mounted a severe negative feedback response, with expression plummeting to ~238–245 FPKM.

Furthermore, exogenous MF triggered a sex-specific suppression of molting signals within the ecdysteroid pathway. The highly active ecdysone receptor *EcR* (ncbi_128690016) in the female hepatopancreas (~5.2–5.9 FPKM) was drastically suppressed to ~0.59–0.72 FPKM at 1000 ng MF, indicating a regulatory shift from molting to reproductive energy allocation. Within the co-activation network, the *E75* nuclear receptor showed remarkable sex-specificity. Its minor transcript (MSTRG.34677) acted as an absolute female-specific switch, stably expressed at 4.2–5.0 FPKM but completely absent in unmanipulated males and intersex individuals. Conversely, the dominant *E75* variant (MSTRG.34676), while abundant in normal sexes (~21–27 FPKM), was abnormally doubled in the intersex control (>42–43 FPKM) and only normalized under high-dose MF intervention.

Finally, downstream reproductive and detoxification effectors exhibited strict physiological boundaries. The vitellogenin receptor *VgR* (ncbi_128690617) was strictly suppressed basally across all phenotypes. However, low-dose MF exclusively activated its expression in females, surging from ~0.17 to 1.13 FPKM, while leaving male and intersex hepatopancreases entirely unresponsive. In contrast, the MF-degrading enzyme *JHE-L* (ncbi_128697980) demonstrated highly conserved pharmacokinetics across all sexes. It maintained full basal expression (~12.1–14.8 FPKM), sharply declined under low-dose MF—dropping to ~6.5–8.1 FPKM in the intersex group to facilitate receptor binding—and rapidly rebounded under toxic high-dose MF (~10.1–13.1 FPKM) to execute robust hormonal clearance.

### 2.4. Sexually Dimorphic Responses to MF Exposure in Normal Males and Females

**Figure 2:**
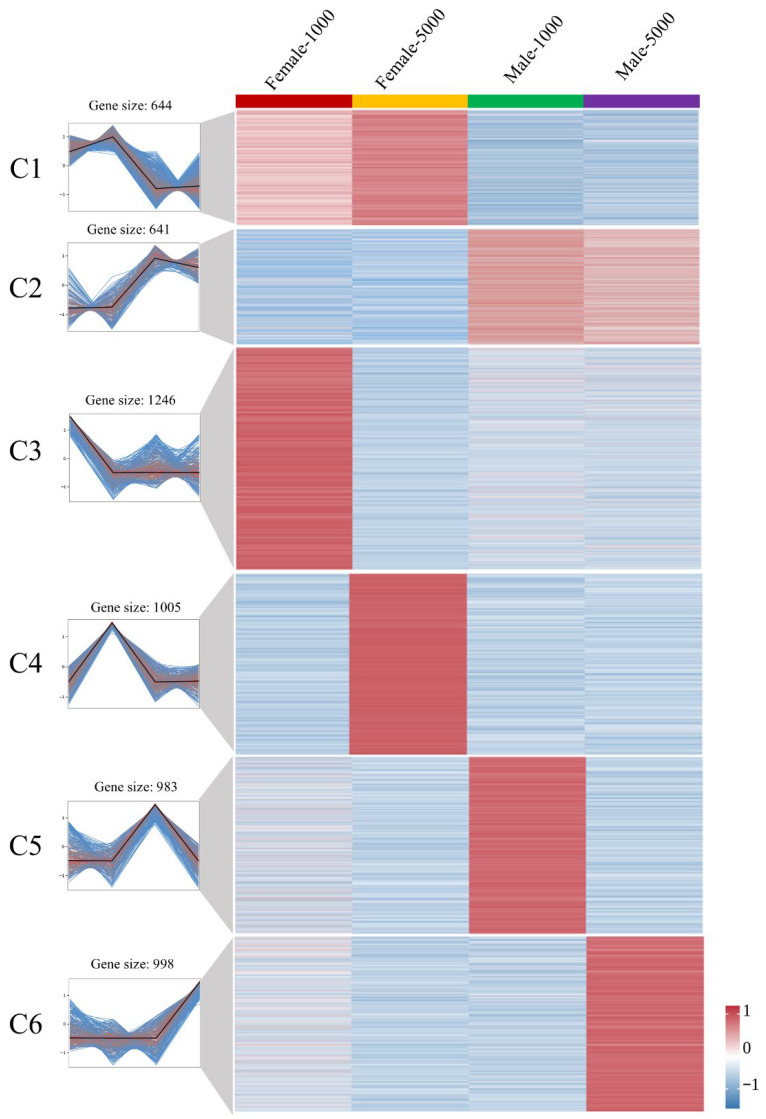
Hierarchical clustering and expression heatmap of DEGs demonstrating sexually dimorphic responses between normal females and males under MF exposure (Clusters C1–C6).

To investigate the different expression patterns between normal female and male crayfish under MF exposure, the DEGs were grouped into six distinct clusters (C1 to C6) based on their expression profiles. The heatmap revealed clear sexually dimorphic responses. Cluster 1 (C1) contained 644 genes that were highly expressed in both 1000 ng and 5000 ng female groups (F-1000, F-5000), but showed low expression in the male groups (M-1000, M-5000). In contrast, Cluster 2 (C2) consisted of 641 genes that exhibited the exact opposite trend, with low expression in females and high expression in males across both doses. Additionally, clusters C3 to C6 represented genes with dose-specific activation, such as C3 (1246 genes) peaking only in F-1000 and C6 (998 genes) peaking only in M-5000 (Figure 2).

**Figure 3:**
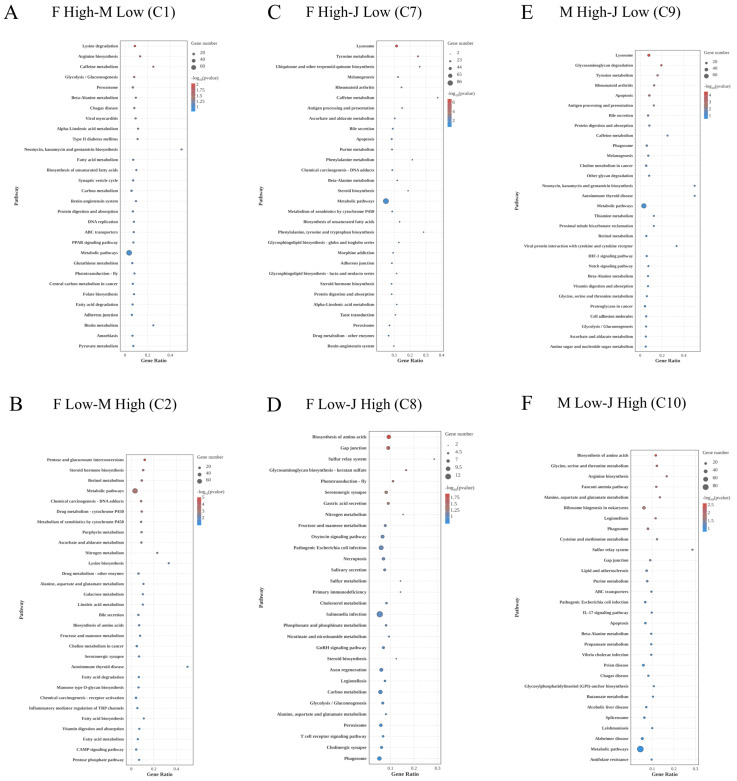
KEGG pathway enrichment analysis of specific differentially expressed gene clusters (C1, C2, C7–C10) across distinct sexual phenotypes following MF exposure. (**A**,**B**) Bubble plots showing enriched pathways for female-biased genes in Cluster 1 (F High-M Low) and male-biased genes in Cluster 2 (F Low-M High). (**C**,**E**) Enriched pathways for genes specifically suppressed in the intersex phenotype compared to normal females (Cluster 7; F High-J Low) and normal males (Cluster 9; M High-J Low). (**D**,**F**) Enriched pathways for genes uniquely upregulated in the intersex phenotype compared to normal females (Cluster 8; F Low-J High) and normal males (Cluster 10; M Low-J High).

To further understand the biological functions driving these sex-specific differences, KEGG pathway enrichment analysis was performed on the C1 and C2 clusters. For the female-biased genes in C1 (F High-M Low), the significantly enriched pathways were mainly associated with basic metabolism and transport. These included Lysine degradation, Arginine biosynthesis, Glycolysis/Gluconeogenesis, and ABC transporters (Figure 3A). Conversely, the male-biased genes in C2 (F Low-M High) were enriched in distinct functional categories. The most prominently enriched pathways included Steroid hormone biosynthesis, Retinol metabolism, Drug metabolism-cytochrome P450, and Metabolism of xenobiotics by cytochrome P450. This indicates a strong male-specific transcriptomic reallocation toward hormone synthesis and enzymatic detoxification of exogenous substances following MF injection (Figure 3B).

### 2.5. The Unique Compensatory and Anti-Apoptotic Architecture of the Intersex Phenotype

**Figure 4:**
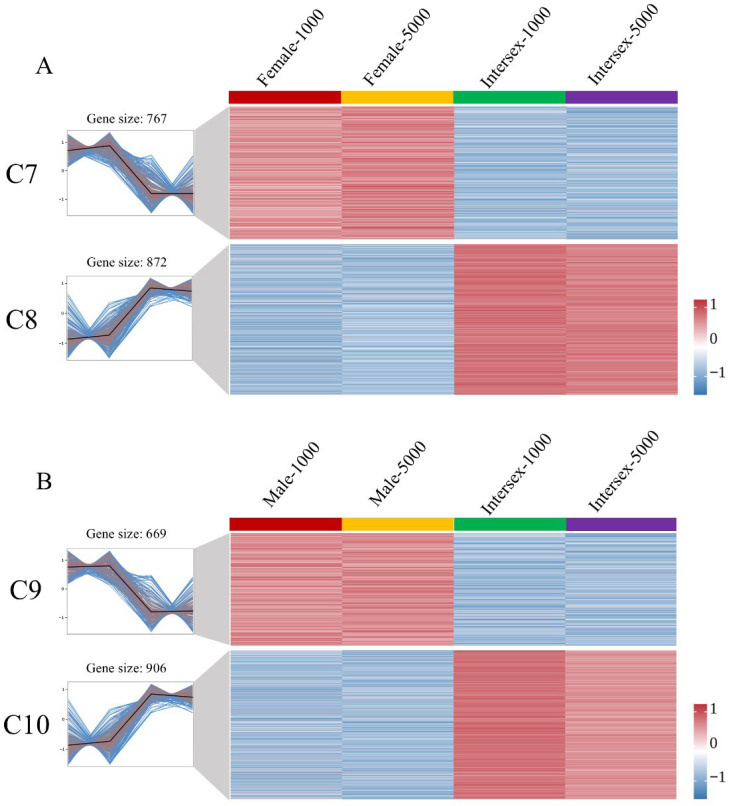
Hierarchical clustering and expression heatmap of DEGs comparing the unique compensatory transcriptomic responses of the intersex phenotype with normal females (**A**) and normal males (**B**) (Clusters C7–C10).

To understand the specific response of intersex crayfish to MF exposure, we compared their gene expression patterns with those of normal females and males. As shown in Figure 4A, the DEGs between normal females and intersex individuals were grouped into two main clusters. Cluster 7 (C7) contained 767 genes with high expression in both female groups (F-1000, F-5000) but low expression in the intersex groups (J-1000, J-5000). Conversely, Cluster 8 (C8) consisted of 872 genes that were down-regulated in females but highly up-regulated in the intersex groups (Table S4). Similarly, the comparison between normal males and intersex crayfish revealed distinct expression patterns (Figure 4B). Cluster 9 (C9) included 669 genes that were highly expressed in normal males but suppressed in intersex individuals. Cluster 10 (C10) contained 906 genes showing low expression in males but high expression in the intersex groups (Table S5).

To explore the biological functions of these specific clusters, KEGG pathway enrichment was performed. Notably, the genes that were lowly expressed in intersex crayfish compared to both females (C7) and males (C9) shared similar enriched pathways. Figure 3C,E show that both the “F High-J Low” (C7) and “M High-J Low” (C9) clusters were significantly enriched in Lysosome and Apoptosis pathways. This suggests that intersex individuals actively suppressed lysosomal degradation and cell death processes under MF exposure. On the other hand, the genes highly expressed in intersex individuals compared to females (C8) and males (C10) also exhibited overlapping functions. As shown in Figure 3D (“F Low-J High”) and Figure 3F (“M Low-J High”), the most significantly enriched pathway in both clusters was Biosynthesis of amino acids. Other shared pathways included Gap junction and Sulfur relay system. This indicates that the intersex phenotype triggered a unique compensatory metabolic strategy, prioritizing amino acid synthesis and cellular communication when challenged with MF.

### 2.6. Co-Expression Network Modules Driven by Gender and Endocrine Disruption

To systematically identify specific gene clusters associated with gender differences and MF exposure, a Weighted Gene Co-expression Network Analysis (WGCNA) was performed. As shown in the cluster dendrogram (Figure S3), the expressed genes were successfully grouped into multiple distinct co-expression modules.

Next, we evaluated the correlation between these generated modules and the specific traits of Gender and MF (Figure S4). The gene significance analysis demonstrated that the purple and royalblue modules had the highest correlation and significance scores with Gender (Figure S4B). On the other hand, the skyblue and blue modules were most significantly associated with the MF treatment (Figure S4C). The detailed expression patterns of these specific modules across all experimental groups were further visualized in the module-sample heatmap (Figure S5).

**Figure 5:**
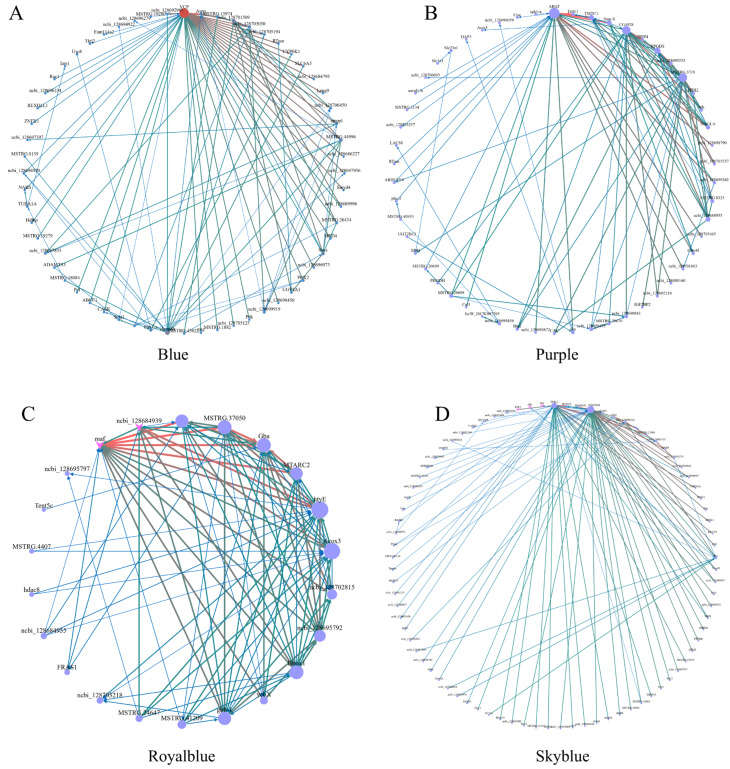
Gene co-expression interaction networks revealing core regulatory hub genes within the key WGCNA modules (Blue, Purple, Royalblue, and Skyblue). In the networks, the size of the nodes (dots/symbols) is proportional to their intra-modular connectivity, visually highlighting the core hub genes. The different colored lines represent the varying weights of the co-expression relationships between the genes. (**A**) Blue Module: This module is primarily driven by methyl farnesoate (MF) dose. The central hub gene, *VCP*, commands the proteotoxic stress response, orchestrating endoplasmic reticulum-associated degradation (ERAD) to manage the translational burden induced by MF. (**B**) Purple Module: A gender-related module featuring *ABAT* and *TMTC1* as central regulatory nodes. (**C**) Royalblue Module: This module is strictly driven by gender and phenotypic dimorphism. The key hub genes, *maf* and *hdac8*, act as central molecular switches for oxidative stress sensing and epigenetic override, respectively, maintaining the masculinized phenotype of intersex individuals. (**D**) Skyblue Module: A module showing high responsiveness to MF exposure, characterized by a dense and highly interconnected regulatory architecture representing the acute transcriptomic shock response.

To discover the core regulatory genes within these highly trait-correlated modules, gene interaction networks were constructed for the Blue, Purple, Royalblue, and Skyblue modules (Figure 5). In the Blue module network (Figure 5A), genes such as *VCP* occupied the central hub position with the highest connectivity. The Purple module network (Figure 5B) highlighted distinct central nodes, including *ABAT* and *TMTC1*. For the Royalblue module (Figure 5C), which is strongly driven by gender, several critical hub genes were identified, notably *maf*, *hdac8*, and *Gba*. Finally, the Skyblue module (Figure 5D), which was highly responsive to MF exposure, displayed a dense and highly interconnected regulatory architecture. These identified hub genes likely act as the central molecular switches orchestrating the sexually dimorphic and stress-compensatory responses to endocrine disruption (Table S3).

### 2.7. qRT-PCR Validation of Hormone Receptor Axis and WGCNA Core Hub Genes

To validate the reliability of the RNA-seq data, five key genes identified from the hormone-receptor axis and WGCNA core modules (*Met*, *Kr-h1*, *VCP*, *hdac8*, and *maf*) were selected for qRT-PCR analysis. As shown in Figure S6, the relative expression patterns of these selected genes across the different sexual phenotypes and MF exposure doses were highly consistent with the FPKM trends derived from the transcriptomic data, demonstrating the high accuracy and reproducibility of our RNA-seq analysis.

## 3. Discussion

Before interpreting the transcriptomic networks, it is essential to clarify the conceptual framework of this study. The intersex phenotype in *C. quadricarinatus* is a naturally occurring, genetically determined condition (WZ genotype), rather than an anomaly induced by environmental contamination. Therefore, the exogenous administration of MF was employed here strictly as a pharmacological probe to challenge distinct sexual backgrounds, rather than as an etiological agent revealing general mechanisms of environmental endocrine disruption.

### 3.1. Genotypic–Phenotypic Conflict-Driven Transcriptomic Hypersensitivity and Evolutionary Adaptation

In the global transcriptomic overview, the most striking discovery is the massive transcriptomic burst exhibited by intersex individuals in response to acute hormonal perturbation. While normal male and female crayfish demonstrated a moderate and controlled transcriptomic response to a 1000 ng dose of MF, intersex individuals initiated a large-scale genomic remodeling, with the number of upregulated genes surging to an astounding 3469. This extreme hypersensitivity profoundly indicates that the intracellular environment of the intersex hepatopancreas operates at a chronically sensitized physiological baseline, a state of elevated allostatic load often observed in aquatic organisms facing persistent internal or environmental stressors [23].

This vulnerability stems from a profound genetic paradox: intersex *C. quadricarinatus* carry a heterogametic female (WZ) genotype, yet they possess male gonopores and function reproductively as males [3]. Operating a female genetic blueprint within a highly masculinized physiological environment—driven by the dominant expression of the insulin-like androgenic gland hormone (IAG)—creates an inherent genotypic–phenotypic conflict [24]. In a normal gonochoristic state, hormonal feedback circuits are strictly defined by the organism’s genotype. However, in the intersex model, the sudden influx of exogenous MF violently disrupts the delicate homeostatic balance between the underlying WZ genetic program and the active male phenotypic machinery, leading to profound endocrine disruption [23]. Consequently, the hepatopancreas is forced into a state of intense transcriptomic activity, activating thousands of genes in a rapid attempt to reallocate metabolic substrates, detoxify the excess hormone, and maintain tissue integrity.

From an evolutionary biology and population ecology perspective, this highly complex transcriptomic remodeling capacity provides the molecular foundation for the survival of intersex individuals in nature. Because these intersexual individuals (WZ males) are functionally reproductive, they are capable of mating with normal WZ females, a process that leads to the natural occurrence of extremely rare homogametic WW females [3]. Ecological modeling of population dynamics suggests that the presence of intersexuals significantly contributes to the short-term growth rate and overall fitness of crayfish populations. The native habitat of *C. quadricarinatus* in Australia is characterized by extreme fragmentation and frequent drought-flood cycles [25]. In such unstable environments, the genetic heterogeneity and robust physiological buffering capacity provided by intersex individuals serve as a critical reproductive buffer. As classically defined in evolutionary developmental biology, such phenotypic plasticity enables species to endure extreme environmental bottlenecks, supporting population restoration when facing extinction threats and aiding species establishment during colonization events into new, isolated habitats [3,26].

### 3.2. Metabolic and Defensive Shunting Strategies in Typical Sexual Dimorphism

In normal males and females, the transcriptomic partitioning of the hepatopancreas in response to MF exposure reveals strict physiological priority boundaries. By analyzing distinct expression profiles (Clusters 1 and 2), our findings demonstrate that the typical sexes deploy diametrically opposed survival and resource allocation strategies when confronted with identical endocrine surges. This dynamic perfectly mirrors the sexually dimorphic pleiotropic effects of MF in crustaceans: acting as a potent gonadotropin to drive vitellogenesis in females, while stimulating allometric growth and reproductive behaviors in males [10,11].

The female-biased transcriptomic routing (Cluster 1) is highly inclined toward energy preservation and the accumulation of reproductive substrates. Specifically, female crayfish exhibited significant enrichments in glycolysis/gluconeogenesis, arginine biosynthesis, and lysine degradation pathways. In decapod crustaceans, the hepatopancreas functions as the central metabolic hub and must mobilize substantial lipid and protein reserves to supply the developing ovary during vitellogenesis [16]. The widespread upregulation of ABC transporters further supports the active transmembrane shuttling of these critical metabolic precursors. Furthermore, the enhancement of the lysine degradation pathway suggests a rapid turnover of the intracellular protein pool, enabling females to meet the immense energetic demands initiated by MF-driven reproductive preparation.

Conversely, the male hepatopancreas (Cluster 2) adopted a highly defensive and steroidogenic posture. The profound enrichment of the cytochrome P450 (CYP) superfamily in drug and xenobiotic metabolism pathways indicates that males primarily perceive the high-titer exogenous MF injection as a pharmacological stressor [27,28]. Consequently, the tissue prioritizes rapid enzymatic detoxification and the clearance of excess hormones to prevent toxicity. Furthermore, the male-specific upregulation of retinol metabolism and steroid hormone biosynthesis pathways points to a complex metabolic crosstalk: while actively degrading the exogenous sesquiterpenoid hormone (MF), the male hepatopancreas is simultaneously remodeling its downstream steroidogenic networks.

This male-biased defensive and steroidogenic routing in *C. quadricarinatus* presents an intriguing interspecies divergence when compared to recent findings in the whiteleg shrimp (*L. vannamei*) [21]. In *L. vannamei*, males exposed to MF predominantly upregulate sugar, lipid, and chitin metabolism (e.g., *CHIT1*) to prepare for molting and structural remodeling, while females prioritize immune responses and miRNA processing. In contrast, the male redclaw crayfish response is heavily skewed toward pharmacological clearance and specific steroid hormone biosynthesis, highlighting distinct, species-specific evolutionary adaptations in endocrine management across different decapod taxa.

### 3.3. Paradoxical Hormone Receptor Dynamics and Negative Feedback Collapse in the Intersex Model

The intracellular genomic action of MF is primarily mediated by a highly conserved signaling cascade driven by the basic helix-loop-helix Per-ARNT-Sim (bHLH-PAS) receptor Methoprene-tolerant (Met) and its downstream early-response transcription factor, Krüppel homolog 1 (Kr-h1) [29,30]. An in-depth transcriptomic dissection of this hormone-receptor axis reveals profound regulatory aberrations within the intersex phenotype, providing a mechanistic core for understanding its genotypic–phenotypic conflict.

In an undisturbed physiological baseline state, the *Met* receptor exhibits moderate expression in normal females and lower expression in males, yet it is nearly completely silenced in intersex individuals. This extremely weak baseline expression likely represents a critical, tissue-level self-protective mechanism. Because the cellular environment of intersex individuals is already dominated by masculinizing factors (such as the insulin-like androgenic gland hormone, IAG) [13,24], downregulating the primary sesquiterpenoid receptor may prevent the unintended amplification of endogenous MF or trace environmental signals, thereby avoiding premature ovotestis activation or developmental disorder. However, when challenged with the acute pharmacological toxicity of a high-dose (5000 ng) MF injection, the intersex group mounted an extreme compensatory rebound, with *Met* expression surging past the levels of normal females. This indicates that while the intersex hepatopancreas attempts to ignore endogenous hormones at baseline, the insurmountable pharmacological pressure forces the receptor machinery back online to initiate emergency downstream target transcription and hormone clearance protocols.

The expression dynamics of the downstream key transcription factor, *Kr-h1*, even more vividly illustrate this endocrine paradox. *Kr-h1* serves as a cornerstone regulator in arthropods, functioning as a universal repressor of metamorphosis and an essential mediator of reproductive homeostasis [29,31]. In the normal female hepatopancreas, *Kr-h1* demonstrated a classic, dose-dependent activation in response to MF. Astoundingly, the intersex control group exhibited a hyperactive *Kr-h1* baseline, with expression levels more than double those of normal females. This intense transcriptional noise suggests that the female (WZ) genome, trapped within a masculinized phenotype, is continuously attempting to force a female reproductive or anti-metamorphic signal through persistent *Kr-h1* overexpression.

When exogenous MF was introduced, *Kr-h1* expression in the intersex individuals did not continue to climb as expected; instead, it experienced a precipitous drop. In molecular endocrinology, this signifies an extreme negative feedback loop. Because the baseline system was already operating at maximum capacity, the sudden influx of a high-titer agonist likely induced severe receptor desensitization or triggered inhibitory transcriptional complexes to prevent lethal metabolic overload. This *Kr-h1* collapse exposes the inherent vulnerability of the intersex endocrine network: lacking a normal physiological buffer to generate a progressive dose–response, the system can only resort to a transcriptional shutdown to avert a complete collapse of cellular homeostasis.

Furthermore, MF exposure triggered a specific boundary reconstruction within the ecdysteroid signaling pathway. The highly active ecdysone receptor (*EcR*) was drastically suppressed in the female hepatopancreas following MF treatment, confirming a strong metabolic antagonism between MF and the molting pathway, which acts to redirect energy from somatic growth toward reproductive preparation [10,17]. Within the co-activation network, the nuclear receptor *E75* demonstrated absolute sexual dimorphism: its minor transcript functioned as a strict switch of females, being stably expressed in females but completely absent in unmanipulated males and intersex individuals. Conversely, the dominant *E75* variant was abnormally doubled in the intersex baseline and was only forcefully normalized under high-dose MF intervention. These receptor dynamics strongly suggest that the intersex model is not merely a superimposition of male and female traits, but rather the operation of a highly strained, easily saturated chimeric endocrine network governed by aberrant feedback mechanisms.

### 3.4. Anti-Apoptotic and Lysosomal Suppression Strategies in the Intersex Hepatopancreas

To survive the severe pharmacological stress induced by the massive influx of MF, the intersex hepatopancreas deploys a highly specialized transcriptomic strategy that sharply contrasts with those of normal males and females. Through comparative analysis (Clusters 7 and 9), our findings reveal that intersex individuals actively and significantly suppressed pathways related to the Lysosome and Apoptosis, which remained highly responsive and elevated in both normal sexes.

In crustacean physiology, the hepatopancreas is the organ most sensitive to environmental, pathogenic, and chemical stressors [15]. Under acute pharmacological challenge, the standard cellular defense response typically involves the hyperactivation of lysosomal autophagy to clear damaged organelles, misfolded proteins, and toxic aggregates. However, if the stressor overwhelms the cellular repair capacity, excessive lysosomal permeability can lead to the release of cathepsins and reactive oxygen species (ROS), ultimately triggering programmed cell death or apoptosis [32]. For normal males and females—whose phenotypes perfectly align with their genotypes—moderate lysosomal activity and controlled apoptosis permit orderly cell turnover and tissue remodeling during MF-induced metabolic shifts.

For intersex individuals, however, the baseline physiological state is already operating under immense biochemical tension due to their inherent WZ/ZZ genetic-phenotypic conflict. When challenged with high-dose MF, the intersex hepatopancreas initiates a massive transcriptomic shock wave, mobilizing over 3000 differentially expressed genes to manage the sudden endocrine overload. If the intersex hepatopancreas allowed standard apoptotic cascades to proceed under such an extreme physiological burden, we hypothesize that unrestrained cell death under such conditions could potentially lead to severe tissue degradation, prompting the observed anti-apoptotic transcriptomic shift.

Therefore, the active downregulation of lysosomal activity and the blockade of apoptotic pathways represent a desperate, yet highly effective, cellular survival strategy unique to the intersex phenotype. By forcefully shutting down programmed cell death, the intersex organism compels its hepatopancreatic cells to continue functioning throughout the acute toxicity phase. This phenomenon is mechanistically reminiscent of specialized anti-apoptotic adaptations observed in some crustaceans that allow them to survive severe environmental insults, energy-limited states [33], or persistent viral infections [34]. In the context of extreme hormonal perturbation, this anti-apoptotic transcriptomic redirection enables the intersex hepatopancreas to maintain its essential glandular structural integrity just long enough for its detoxification networks to clear the excess MF.

### 3.5. Compensatory Metabolic and Communication Architecture Under Extreme Stress: Amino Acids, Sulfur Relay, and Gap Junctions

Because the intersex hepatopancreas actively shuts down traditional autophagic and apoptotic clearance pathways, it must rely on a series of alternative biochemical networks to manage the massive oxidative stress and proteotoxicity generated by the influx of MF. The transcriptomic data clearly demonstrate that this compensation is achieved through the intense, specific upregulation of the Biosynthesis of amino acids, Sulfur relay system, and Gap junction pathways (Clusters 8 and 10), which distinguishes the intersex response from that of normal males and females.

#### 3.5.1. Amino Acid Biosynthesis as an Antioxidant Substrate Pump

The extreme enrichment of amino acid biosynthesis pathways is a hallmark of metabolic reconfiguration in organisms enduring severe environmental or hormonal stress. During acute physiological stress, crustaceans rapidly mobilize free amino acid pools, channeling them into the tricarboxylic acid (TCA) cycle for energy maintenance and utilizing them to synthesize critical stress-response proteins [35]. In this compensatory network, specific newly synthesized amino acids serve as direct precursors for glutathione, the primary intracellular antioxidant [36]. By prioritizing de novo amino acid synthesis, the intersex hepatopancreas ensures a continuous supply of reducing equivalents. This metabolic shift is vital for neutralizing the explosive generation of reactive oxygen species (ROS) triggered by the large-scale transcriptomic and metabolic reorganization necessary to handle the MF surge, a xenobiotic-driven oxidative phenomenon widely documented in aquatic invertebrates [36,37].

#### 3.5.2. Deep Detoxification and Translational Control via the Sulfur Relay System

Closely linked to the surge in amino acid synthesis is the highly specific activation of the Sulfur relay system. This evolutionarily ancient and highly conserved biochemical pathway is responsible for the precise transfer of sulfur atoms from donor molecules to specific intracellular targets. It serves as an essential driver for the 2-thiolation of tRNA wobble bases and for ubiquitin-like protein modifications (such as the URM1 pathway) [38].

In aquatic invertebrates, the activation of the sulfur relay system has been identified as a critical adaptive response to acute pharmacological overload and xenobiotic chemical stress, significantly enhancing cellular tolerance and antioxidant capacity. A highly coordinated metabolic shift toward sulfur assimilation is a proven fundamental defense mechanism against severe oxidative stress, directly linking methionine/cysteine metabolism to global redox buffering [39]. In the intersex hepatopancreas, the hyperactivation of this system indicates an extraordinarily high demand for sulfur-containing cofactors. This mechanism not only maintains redox balance under extreme oxidative tension but also ensures the fidelity of ribosomal translation under a massive transcriptional load via tRNA modification [38]. Furthermore, given the deep evolutionary homology between prokaryotic sulfur-relay mechanisms and eukaryotic ubiquitination pathways, the heightened activity of this system strongly suggests that intersex cells are exploiting ancient ubiquitin-like modifications to tag misfolded proteins for targeted degradation. This allows the cell to maintain proteostasis without needing to trigger global autophagic cell death.

#### 3.5.3. Gap Junction-Driven Metabolic Syncytium

To coordinate this massive metabolic and defensive shift at the whole-organ level, the intersex hepatopancreas drastically upregulated transcripts associated with gap junctions. In invertebrates, including crustaceans, gap junctions are primarily composed of innexins, which form direct intercellular communication channels allowing the rapid shuttling of ions, second messengers, and metabolic intermediates between adjacent cells [40,41].

Previous physiological studies have demonstrated that ionic coupling and gap junction activity in the crustacean hepatopancreas significantly increase during hormonal signaling and molting events. Under the extreme pharmacological pressure of an MF influx, the explosive upregulation of gap junction genes effectively transforms the intersex hepatopancreas into a giant metabolic syncytium. This tight intercellular coupling allows the most severely damaged or overwhelmed cells to offload toxic metabolic intermediates to neighboring, healthier cells while simultaneously distributing critical survival signals and antioxidant precursors throughout the tissue [41]. Crucially, gap junctional communication is a recognized double-edged sword in tissue stress; however, controlled coupling acts as a physiological shock absorber, actively dissipating lethal stress signals [42]. By deploying this massive intercellular network, the intersex organism may attempt to limit localized cellular necrosis and mitigate the risk of cascading tissue failure, thereby safeguarding the overall glandular integrity of the hepatopancreas.

### 3.6. Core Regulatory Network Hubs at the Intersection of Proteostasis, Oxidative Stress, and Epigenetics

Weighted Gene Co-expression Network Analysis (WGCNA) effectively distilled the massive and complex transcriptomic datasets into highly aggregated functional modules, precisely identifying the core regulatory hub genes that drive sexual differentiation and the physiological response to MF exposure. Among these, *VCP*, *maf*, and *hdac8* emerged as central nodes, providing a high-resolution molecular map of how the crustacean hepatopancreas executes survival and phenotypic maintenance strategies under severe endocrine tension.

#### 3.6.1. *VCP* Commands the Proteotoxic Stress Response

Valosin-containing protein (VCP, also known as p97 or Cdc48) occupies the absolute core hub position within the Blue module, which is highly responsive to MF exposure. VCP is an abundant and highly conserved AAA+ ATPase that plays an irreplaceable role in maintaining protein and organelle homeostasis. Its primary cellular function is to extract polyubiquitinated, misfolded proteins from the endoplasmic reticulum (ER) membrane and escort them to the proteasome for degradation, a process known as ER-associated degradation (ERAD) [43].

When the hepatopancreas of *C. quadricarinatus* is exposed to high-dose MF, the cells are forced to synthesize a massive wave of detoxification enzymes, transmembrane transporters, and metabolic regulators in a very short period. This sudden translational burst places an extreme folding burden on the ER, inevitably leading to the accumulation of toxic, misfolded proteins. In decapod crustaceans, unresolved ER stress in the hepatopancreas acts as a primary trigger for the unfolded protein response (UPR) and subsequent apoptotic cascades [44]. The centralized explosion of *VCP* in the transcriptomic network reveals that the organism’s frontline defense against MF toxicity is not merely chemical detoxification, but rather robust protein quality control. Acting as a powerful molecular motor, *VCP* unravels toxic protein aggregates, ensuring that the translational machinery is not jammed by its own waste [43,44]. This vital function buys precious execution time for the compensatory amino acid and sulfur relay pathways discussed previously, emphasizing that surviving severe endocrine disruption is fundamentally a battle to maintain cellular proteostasis.

#### 3.6.2. *Maf* Acts as a Dual Sensor for Oxidative Stress and Dimorphic Differentiation

The transcription factor *maf* was identified as a critical hub in the Royalblue module, which is strictly driven by gender and phenotypic bias. The Maf family consists of basic leucine zipper (bZIP) transcription factors that regulate target gene expression by forming homo- or heterodimers, playing a pivotal role in cellular differentiation and the response to oxidative and xenobiotic stress [45]. In arthropods, particularly within the decapod hepatopancreas, small Maf proteins are essential co-factors for the *Nrf2* signaling pathway, regulating antioxidant response elements (AREs) to orchestrate the mass overexpression of detoxification enzymes during chemical exposure [46].

In the physiological context of the *C. quadricarinatus* hepatopancreas, *maf* exerts a dual integration function. First, as a gender-correlated hub, our transcriptomic data reveals a stark dimorphism: *maf* expression is stably maintained in normal females but profoundly suppressed in both normal males and intersex individuals. This indicates that in the intersex hepatopancreas, the masculinized phenotypic machinery successfully overrides the WZ genotypic baseline to enforce a male-typical *maf* suppression. Second, this inherent suppression directly links sexual phenotype to oxidative stress management. Normal females maintain a stable *maf* baseline, equipping them with a robust, preemptive Nrf2/ARE antioxidant buffering capacity. In contrast, intersex individuals lack this steady-state *maf*-mediated defense. When challenged with the massive oxidative imbalance triggered by an acute MF influx, they are uniquely disadvantaged and cannot rely on this conventional axis. This inherent vulnerability elegantly explains the molecular origins of the vast compensatory antioxidant networks—such as the aforementioned surge in de novo amino acid biosynthesis and sulfur relay systems—uniquely mobilized by the intersex phenotype to survive the severe pharmacological stress.

#### 3.6.3. *Hdac8* Mediates Epigenetic Override and Chromatin Lockdown

Perhaps the most profoundly significant hub gene within the gender-driven Royalblue module is histone deacetylase 8 (*hdac8*). Epigenetic modifications, particularly histone acetylation and deacetylation, act as master switches controlling global chromatin accessibility and transcriptional activity, enabling large-scale phenotypic remodeling without altering the underlying DNA sequence [47]. HDAC enzymes remove acetyl groups from histones, generally leading to chromatin condensation and the suppression of specific gene networks.

The transcriptomic baseline of *hdac8*—which is stably maintained in normal females but profoundly suppressed in both normal males and intersex individuals—provides a compelling epigenetic explanation for the intersex phenomenon itself. Intersex redclaw crayfish possess a female WZ genotype but exhibit functional male physiological structures. To achieve this cellular deception, the organism must fundamentally escape its own genetic blueprint. In normal females, high HDAC8 activity likely acts as the epigenetic enforcer, deacetylating chromatin to lock male-specific developmental networks into a transcriptionally inactive heterochromatin state. Therefore, by aggressively downregulating *hdac8* to mirror the normal male baseline, the intersex hepatopancreas successfully lifts this epigenetic suppression, allowing ZZ-equivalent masculinized expression networks to remain forcefully open while arresting ovarian tissue in a pre-vitellogenic form.

Crucially, it is well-established that epigenetic regulators like HDACs are highly sensitive to massive hormonal shifts and pharmacological challenges, which can induce permanent phenotypic shifts by forcing chromatin remodeling [48,49]. For the intersex crayfish, maintaining the suppressed state of *hdac8* is a delicate act of epigenetic tension required to sustain the masculinized phenotype. When injected with a massive dose of MF, this fragile equilibrium is abruptly destabilized. Under these extreme conditions, stress-induced fluctuations in *hdac8* expression directly dictate whether thousands of chromatin regions are erroneously forced open or emergency-locked in response to the disruption. Thus, *hdac8* represents the vital molecular bridge connecting the organism’s hardcoded genetic blueprint (WZ), its plastic sexual phenotype, and its real-time transcriptomic shock response to exogenous hormonal perturbation.

### 3.7. Broad Implications for Evolutionary Biology and Aquaculture

The sexually dimorphic transcriptomic regulation exhibited by the *C. quadricarinatus* hepatopancreas in response to MF exposure not only expands our fundamental understanding of crustacean endocrine plasticity but also carries profound implications for evolutionary ecology and aquacultural biotechnology.

From a macro-evolutionary perspective, the extremely robust, counter-intuitive anti-apoptotic compensatory mechanisms deployed by the intersex phenotype provide a compelling explanation for why these unique WZ males survive in nature. In their native Australian habitats, *C. quadricarinatus* populations frequently face extreme environmental instability, characterized by highly fragmented environments and severe drought-flood cycles. Under such harsh conditions, the intersex organism’s ability to sacrifice normal cellular turnover programs in favor of preventing catastrophic organ collapse affords it a distinct survival advantage. As widely discussed in modern evolutionary biology, such extreme phenotypic plasticity and physiological buffering capacity can act as a crucial bridge for evolutionary innovation, allowing populations to persist under severe environmental fluctuations [50]. Because these intersexual individuals act as functionally reproductive males, their survival theoretically enables them to mate with normal WZ females, giving rise to homogametic WW females. Prior studies have confirmed that these WW females are fully viable and fertile. Population modeling suggests that their introduction shifts the overall sex ratio toward females, which can temporarily increase the population growth rate and aid in demographic recovery during environmental bottlenecks [3]. However, it is crucial to emphasize that the current study relies exclusively on transcriptomic data and does not provide direct functional or reproductive assessments. The profound transcriptomic mobilization observed in the intersex hepatopancreas merely uncovers a molecular buffering capacity. Therefore, these evolutionary mechanisms—along with the subsequent aquaculture implications, such as leveraging specific network hubs (e.g., *hdac8*, *maf*) for non-invasive monosex breeding technologies—should be strictly interpreted as hypothesis-generating frameworks. Extensive in vivo functional validations, such as gene knockdown and long-term reproductive trials, are required before these transcriptomic signatures can be definitively linked to population-level evolutionary fitness or commercial aquaculture applications.

From the standpoint of biotechnology and commercial aquaculture, the establishment of monosex cultures (such as all-male populations) remains a primary objective to maximize yield and economic efficiency in decapod farming, given that males typically exhibit superior growth rates and larger body sizes [14,51]. Traditional methods to manipulate crustacean reproduction and sex differentiation have frequently relied on blunt physical interventions, such as unilateral eyestalk ablation to remove the source of inhibiting neuropeptides. However, these approaches are increasingly scrutinized for their systemic physiological impacts and animal welfare concerns. Fortunately, modern biotechnological approaches, such as the successful mass production of all-male *Macrobrachium rosenbergii* populations via the generation of neo-females, have proven that non-surgical endocrinological manipulation is highly viable at a commercial scale [52].

The underlying molecular mechanisms elucidated in this study offer a new repertoire of high-precision intervention targets to further this goal. Specifically, the epigenetic switch mediated by *hdac8*, the oxidative stress control orchestrated by *maf*, and the highly vulnerable negative feedback loop of *Kr-h1* present highly specific nodes for manipulation. Future research leveraging targeted inhibitors or advanced gene-silencing techniques like RNA interference (RNAi)—which has already been successfully utilized to manipulate the insulin-like androgenic gland hormone (IAG) in this species [13], as well as to replace eyestalk ablation by silencing gonad-inhibiting hormones in shrimp [53]—could allow for the precise directional control of sexual differentiation trajectories. Exploiting these core regulatory hubs could ultimately facilitate the development of advanced, non-invasive monosex breeding technologies without triggering the global toxicity associated with broad-spectrum endocrine disruption.

## 4. Materials and Methods

### 4.1. Experimental Animals and Acclimation

The redclaw crayfish (*Cherax quadricarinatus*) used in this study (3-month-old juveniles, body weight: 7.15 ± 0.48 g) were obtained from Hengzhao lanlong (Guangdong) Aquatic Products Co., Ltd. (Jiangmen, Guangdong, China). Healthy, intact individuals (Individuals were considered healthy and intact if they exhibited normal feeding behavior, could actively and rapidly escape from the collection net, and showed no external injuries or missing appendages) in the intermolt stage were selected for the experiment. The intermolt stage (Stage C) was determined non-invasively by observing the setal development of the pleopods under a light microscope, characterized by the absence of epidermal retraction (apolysis) and the presence of fully formed internal cones within the setae [54]. The physiological phenotypes of the crayfish were strictly identified based on their external morphological characteristics: normal females (F) possess a pair of gonopores at the base of the third pereiopods; normal males (M) possess a pair of genital papillae at the base of the fifth pereiopods; and intersex individuals (J) simultaneously possess both female gonopores and male papillae. To ensure absolute accuracy in classification, we further incorporated secondary sexual characteristics and anatomical verification. Intersex individuals were identified by the simultaneous presence of both sets of gonopores/papillae and the distinct red patches on the outer margin of the chelipeds (a male-specific trait). Subsequent anatomical dissection of representative individuals confirmed that while normal females and males possessed typical ovaries and testes, respectively, intersex individuals exhibited a unique “ovotestis” structure, characterized by functional testicular tissues accompanied by vestigial or arrested ovarian tissues [55]. This multi-level identification approach provided a 100% consistent classification across all experimental groups. Three experimental groups were established for each sex category, with 10 individuals per group receiving injections, resulting in a total sample size of 90 individuals. Prior to the formal experiment, all crayfish were acclimated in recirculating glass aquaria (130 cm × 50 cm × 60 cm; total volume: 390 L) equipped with a biological filtration system for 14 days. To ensure optimal physiological conditions and minimize social stress, each tank was stocked with 30 individuals of a single sex (stocking density: ~46 individuals/m^2^). Multiple shelters, including PVC pipes and mesh structures, were provided in each tank to minimize aggressive interactions and cannibalism. During the acclimation period, the water temperature was maintained at 27 ± 1 °C, dissolved oxygen was kept at >6.0 mg/L, and the photoperiod was set to 12 L:12 D. To maintain optimal environmental stability, key water quality parameters were strictly controlled: pH was maintained at 7.5 ± 0.3, total ammonia nitrogen was kept below 0.05 mg/L, and nitrite levels were maintained below 0.01 mg/L. The biological filtration system ensured efficient nitrification. The crayfish were fed a commercial sinking pelleted diet specifically formulated for freshwater prawns, manufactured by Haida Group Co., Ltd., Jieyang, China. The proximate composition of the diet, as provided by the manufacturer, was approximately: 46% crude protein, 8% crude lipid, 15% crude ash, and 5% crude fiber. Uneaten feed and feces were siphoned out the following day to maintain water quality.

### 4.2. Hormone Treatment and Tissue Collection

Following the acclimation period, a simple random sampling procedure was employed to allocate the crayfish. Given the highly uniform body weight of the selected juveniles (7.15 ± 0.48 g), healthy individuals from the three phenotypic groups (Female, Male, and Intersex) were blindly captured from their respective holding tanks using a hand net. Crucially, each phenotypic group was independently subdivided into three distinct treatment conditions: the control group (CON), the low-dose exposure group (1000 ng), and the high-dose exposure group (5000 ng). This fully crossed design resulted in 9 independent experimental groups (3 phenotypes × 3 treatments). Specifically, 10 individuals from each phenotypic group were allocated to each treatment. This blind-catching method was utilized to actively prevent any subjective human selection bias regarding animal size or activity. The control groups were injected with 50 µL of PBS, while the two experimental groups were injected with fixed absolute doses of 1000 ng and 5000 ng of MF (Amyjet, Wuhan, China) in 50 µL of PBS per individual, respectively. Due to the lipophilic nature of MF, a high-concentration stock solution was initially prepared by dissolving the MF standard in absolute ethanol and stored at −20 °C in the dark to maintain chemical stability. Immediately prior to injection, the working solutions were freshly prepared by diluting the stock solution with sterilized PBS on ice. The final concentration of ethanol in the injected solutions was rigorously maintained below 0.1% (*v*/*v*) to preclude any solvent-related physiological artifacts. Correspondingly, the control group (CON) received 50 µL of PBS containing the exact same trace volume of ethanol as a vehicle control. Because the selected crayfish cohort possessed highly uniform body weights, doses were standardized per individual rather than dynamically adjusted for body weight. This standardized approach was utilized to ensure consistent total hormonal exposure and to minimize micro-volumetric handling errors during injection. The injection was administered into the abdominal cavity at the base of the third pereiopod using a 50 µL microinjection syringe (Hamilton, Reno, NV, USA). To eliminate potential confounding effects of circadian rhythms on gene expression, all injections across all experimental and control groups were strictly performed within a continuous two-hour window in the morning (9:00 AM to 11:00 AM). At 24 h post-injection, all experimental crayfish were subjected to standardized cold anesthesia to minimize handling stress and prevent acute transcriptomic alterations. Specifically, the crayfish were placed on crushed ice for approximately 5–10 min until a complete loss of the righting reflex and cessation of spontaneous movement were observed. Following successful anesthetization, three individuals were randomly selected from each of the 9 experimental groups for transcriptomic analysis. The hepatopancreas tissues were rapidly dissected, snap-frozen in liquid nitrogen immediately upon collection. To ensure an aseptic environment and maximize the quality of the transcriptomic samples, a stringent cleansing protocol for surgical instruments was implemented. After sampling each individual, the scissors and forceps were meticulously disinfected with 75% eqthanol and treated with an RNase decontamination solution (RNaseZap, Thermo Fisher Scientific, Waltham, MA, USA) to eliminate potential cross-contamination and minimize RNase interference. All tissues were subsequently stored in a −80 °C ultra-low temperature freezer for subsequent total RNA extraction. To minimize technical variance, all experimental procedures were performed by a single experienced operator. This sampling strategy yielded three biological replicates per group, totaling 27 independent samples.

### 4.3. RNA Extraction, Library Construction, and Sequencing

Following a brief storage period of less than 48 h at −80 °C, total RNA was extracted from the hepatopancreas tissues. Given the exceptionally high levels of endogenous RNases and lipids inherently present in the hepatopancreas, a combined TRIzol-column protocol was deliberately utilized to maximize RNA integrity and purity. Specifically, the standard lysis buffer from the commercial kit was bypassed. Instead, tissues were initially homogenized and lysed using TRIzol Reagent (Invitrogen, Waltham, MA, USA), which rapidly deactivates RNases and partitions lipids during chloroform phase separation. Subsequently, the RNA-containing aqueous phase was processed using the Eastep Super Total RNA Extraction Kit (Promega, Shanghai, China) for downstream purification, including on-column DNase I treatment and final washing, according to the manufacturer’s protocol. Crucially, no sample pooling was performed during this process. Each of the 27 biological replicates (3 replicates per group × 9 groups) was derived from the hepatopancreas of a single, independent individual. Following extraction, preliminary RNA purity and concentration were measured using a NanoDrop 2000 spectrophotometer (Thermo Fisher Scientific, Waltham, MA, USA), and gross degradation was checked via 1% agarose gel electrophoresis. To meet the stringent requirements for high-throughput sequencing, the precise RNA integrity was systematically quantified using an Agilent 2100 Bioanalyzer (Agilent Technologies, Santa Clara, CA, USA). Only premium-quality RNA samples meeting the strict criteria of OD260/280: 1.8~2.0, OD260/230: ≈2.2, and an RNA Integrity Number (RIN) > 7.0 were utilized for subsequent library construction.

Following RNA quality assessment, sequencing libraries were generated using the Hieff NGS^®^ Ultima Dual-mode mRNA Library Prep Kit (Yeasen Biotechnology, Shanghai, China) in strict accordance with the manufacturer’s instructions without any modifications. Briefly, intact mRNA was purified from the total RNA pool using poly-T oligo-attached magnetic beads. The enriched mRNA was then fragmented and reverse-transcribed into first-strand cDNA using random hexamer primers and reverse transcriptase, followed by second-strand cDNA synthesis. After end repair and adenylation of the 3′ ends, specific DNA sequencing adapters were ligated to the fragments. To select cDNA fragments of the appropriate length and remove unligated adapters, the products were purified using Hieff NGS^®^ DNA Selection Beads (Yeasen, Shanghai, China). Finally, the adapter-ligated fragments were enriched by PCR amplification. Prior to sequencing, the constructed cDNA libraries were subjected to stringent quality control. The absolute library concentration was accurately quantified using a Qubit fluorometer (Thermo Fisher Scientific, Waltham, MA, USA). Furthermore, the fragment size distribution and the absence of adapter dimers were rigorously verified using the Agilent 2100 Bioanalyzer with a High Sensitivity DNA Assay Kit (Agilent Technologies, USA). Only premium libraries exhibiting a single, distinct peak at the expected target insert size (without adapter dimer contamination) and meeting the strict concentration thresholds were pooled and sequenced on the Illumina NovaSeq X Plus platform (Illumina, San Diego, CA, USA) in paired-end 150 bp (PE150) mode. To ensure high-resolution transcriptome coverage and the accurate detection of low-abundance genes, a stringent sequencing depth was applied, yielding a minimum of 6 Gb of clean data (approximately 40 to 55 million paired-end reads) for each independent biological replicate. The finalized and quality-checked cDNA libraries were sequenced on the Illumina NovaSeq X Plus platform (Illumina, San Diego, CA, USA) to generate paired-end reads. The sequencing services were provided by Guangzhou Gene Denovo Co., Ltd., Guangzhou, China.

### 4.4. Data Quality Control and Mapping

To ensure the accuracy of downstream analyses, the raw reads generated from sequencing were subjected to rigorous quality control. Clean reads were obtained by strictly applying the following filtering criteria: (1) removing reads containing adapter sequences; (2) removing reads containing more than 10% unknown nucleotides (N); and (3) removing low-quality reads, which were defined as reads wherein >50% of the bases possessed a Phred quality score of Q ≤ 20. The resulting quality control metrics were computationally validated using fastp software (v0.18.0). The proportion of Clean Data was calculated as the ratio of total clean reads to total raw reads, while the adapter contamination rate was calculated as the ratio of adapter-containing reads to total raw reads. Quality control metrics confirmed that the proportion of Clean Data reached 99.99%, with the adapter contamination rate strictly controlled at approximately 0.01%. Concurrently, the Q20, Q30, and GC content were calculated using fastp (v0.18.0) to evaluate the overall sequencing quality. Subsequently, the high-quality clean reads were mapped to the recently published chromosome-level reference genome of *C. quadricarinatus*. This high-quality reference genome, which was assembled by integrating Nanopore and PacBio long-read sequencing techniques, along with its corresponding annotation files, was acquired from the China National GeneBank DataBase (CNGBdb) under the accession number CNP0005505 (DOI: 10.26036/CNP0005505; NCBI BioProject: PRJNA904538) [56]. To accurately quantify gene expression without the interference of introns, the reference mRNA sequences (reference transcriptome) were extracted based on the genome annotation files. Subsequently, the high-quality clean reads were mapped to this reference transcriptome utilizing Bowtie2 (v2.2.5), which was invoked as the internal alignment engine by the RSEM software package (v1.3.3).

### 4.5. Differential Expression and Clustering Analysis

Following the Bowtie2 alignment against the reference transcriptome, the read counts mapped to each gene were precisely quantified using the RSEM software package with default parameters for paired-end data. Post-quantification, the analytical pipeline was strictly bifurcated based on the statistical requirements. To evaluate the overall gene expression levels and to facilitate downstream visualizations (such as hierarchical clustering and heatmaps), the read counts were normalized using the FPKM method. However, for the rigorous statistical identification of differentially expressed genes, the unnormalized raw read counts were utilized as the direct input for the DESeq2 package in R-4.3.1. Within DESeq2, the expression data were normalized using the built-in median-of-ratios method to estimate size factors and account for differences in sequencing depth. To evaluate the biological reproducibility of the replicates and visualize the global transcriptomic variance across different phenotypes and treatments, Principal Component Analysis (PCA) was performed based on the normalized expression profiles utilizing the prcomp function in R (or the integrated PCA function within the DESeq2 package). Differential expression analysis was performed by fitting the count data to a negative binomial generalized linear model (GLM), and statistical significance was evaluated using the Wald test. To control the false discovery rate (FDR) for multiple testing, the resulting *p*-values were adjusted using the Benjamini–Hochberg procedure. The threshold for identifying differentially expressed genes (DEGs) was set at |log2(Fold Change)| ≥ 1 and an adjusted *p*-value (or FDR) < 0.05. The distribution of DEGs was visualized using Venn diagrams and volcano plots generated via the Omicsmart online platform (Gene Denovo Biotechnology Co., Ltd., Guangzhou, China, https://www.omicsmart.com/ [accessed on 14 December 2025]). To decipher the distinct gene expression patterns among females, males, and intersex individuals under MF exposure, the DEGs were subjected to trend clustering analysis utilizing the Mfuzz package in the R environment. Unlike traditional clustering methods, Mfuzz employs the Fuzzy C-Means (FCM) clustering algorithm, which is highly robust for capturing representative expression trends across multiple experimental groups. Prior to the FCM clustering, a rigorous data filtering and scaling approach was applied via the Omicsmart platform. The fold changes in gene expression between the treatment groups and their respective controls were calculated. Genes exhibiting a fold change > 2 in at least one comparison group were retained, and their log2(Fold Change) values were utilized as the input matrix for the trend analysis. The number of clusters (e.g., resulting in clusters C1–C10) was defined by the FCM algorithm parameters to optimally partition the genes into distinct, coordinated expression profiles representing their specific response trajectories to the MF endocrine disruption. Expression heatmaps were generated utilizing the Omicsmart platform (pheatmap package in R). For this analysis, the expression data (FPKM values) were first standardized using Z-score normalization across samples to ensure comparable expression trends. Subsequently, the hierarchical clustering was executed using the Euclidean distance metric to measure similarity, coupled with the Complete linkage method to construct the dendrograms and partition the genes into distinct expression clusters.

### 4.6. Functional Enrichment Analysis and Weighted Gene Co-Expression Network Analysis (WGCNA)

To elucidate the primary biological functions and metabolic pathways associated with the DEGs within specific clusters, KEGG pathway enrichment analysis was performed. For the hypergeometric test calculations, the background gene set (the universe) was strictly defined as all expressed genes detected in our transcriptomic dataset that possessed valid KEGG annotations. The computation was executed using the clusterProfiler package in R. To rigorously control the false discovery rate (FDR) and account for multiple testing, the calculated *p*-values were systematically adjusted. A KEGG pathway with a Q-value (FDR-adjusted *p*-value) < 0.05 was strictly defined as significantly enriched. Following the unbiased global enrichment analysis across all DEG clusters, the selection of specific pathways for detailed downstream visualization and biological interpretation was strictly data-driven. Pathways demonstrating the highest statistical significance and most profound relevance to the distinct phenotypic responses—specifically those governing basal metabolism, steroid biosynthesis, lysosomal activity, apoptosis, amino acid biosynthesis, and gap junctions—were prioritized to decode the mechanism of MF endocrine disruption.

To systematically identify co-expression modules highly correlated with specific traits, namely Gender and MF Dose a weighted gene co-expression network was constructed using the WGCNA package in R. Initially, to eliminate uninformative background noise and reduce computational burden, genes with low expression levels—strictly defined as those with an FPKM < 2—were filtered out prior to network construction. A gene co-expression similarity matrix was then calculated utilizing the Pearson correlation method to quantify the linear relationships between all retained genes. Subsequently, soft-thresholding power of β = 8 was selected to transform the similarity matrix into an adjacency matrix. This specific power was determined based on the scale-free topology criterion, where β = 8 was the lowest power that enabled the fit index R^2^ to reach the high-stringency threshold of 0.90, thereby ensuring that the resulting network approximated a scale-free topology while preserving maximum biological connectivity. Gene modules were identified utilizing the dynamic tree cut algorithm based on the topological overlap matrix (TOM). To ensure the robustness of the identified modules, the minimum module size was strictly set to 50 genes (minModuleSize = 50), and the sensitivity parameter was set to 2 (deepSplit = 2). Finally, to avoid over-segmentation and consolidate highly correlated modules, a module eigengene clustering was performed, and closely related modules were merged using a height cut-off of 0.25 (mergeCutHeight = 0.25, corresponding to a module correlation threshold of 0.75). The Pearson correlation coefficients between these final merged module eigengenes (MEs) and the phenotypic traits (Gender and MF Dose) were calculated to screen for the core responsive modules. To perform this trait-module correlation analysis, the categorical trait “Gender” was transformed into a binary matrix using dummy coding (where the target phenotype was coded as 1 and all others as 0). Simultaneously, the “MF Dose” trait was encoded as a continuous numeric variable based on the absolute injection concentrations (0, 1000, and 5000 ng). Concurrently, the statistical significance of these correlations was determined by calculating the Student asymptotic *p*-values. A module-trait relationship was strictly defined as statistically significant when the *p*-value was < 0.05. Only these significantly correlated modules were selected for downstream key gene (hub gene) extraction. Within these critical modules, the intra-modular connectivity (KME, or Module Membership) for each gene was calculated to evaluate its centrality and functional importance in the network. To rigorously identify the key functional hub genes, a stringent selection criterion was applied: only genes exhibiting a high intra-modular connectivity of KME > 0.8 were defined as hub genes. These identified hub genes, representing the core expression trends of their respective modules, were then subjected to further functional annotation and visualization. To construct the interaction networks, the edge data connecting these genes were extracted from the Topological Overlap Matrix (TOM). To ensure a focused representation of the most robust biological interactions, the network was filtered by retaining only the top 100 relationship pairs with the highest connectivity weights. The resulting high-confidence interaction network was subsequently visualized using Cytoscape software (v3.9.1).

### 4.7. qRT-PCR Validation

Total RNA was reverse-transcribed into cDNA using the M-MLV Reverse Transcriptase Kit (Invitrogen, Waltham, MA, USA) according to the manufacturer’s instructions. All RNA and cDNA samples were stored in a −80°C freezer. The expression levels of key hub genes (*Met*, *Kr-h1*, *VCP*, *hdac8*, and *maf*) were evaluated. *β-Actin* (MN396754.1) was used as reference gene. The primer sequences for target and reference genes are listed in Table S6. Primers were designed based on conserved sequences in *C. quadricarinatus*, ensuring specificity and amplification efficiency. Real-time fluorescent quantitative PCR (qRT-PCR) was performed using a Step One Plus Real-time PCR system (Applied Biosystems, Foster City, CA, USA). The reaction mixture contained 1 μL (50 ng/μL) of cDNA, 10 μL of 2×SYBR Green Premix (Universal, TIANGEN Biotech Co., Ltd., Beijing, China), 1 μL (10 pmol/μL) of each primer, and 7 μL of ddH_2_O, with a final volume of 20 μL. The qPCR protocol was as follows: 95 °C pre-denaturation for 30 s; 35 cycles of 95 °C denaturation for 40 s, 60 °C annealing for 45 s, and 72 °C extension for 30 s; followed by a final 72 °C extension for 10 min. Gene expression levels were calculated using the 2^−ΔΔCt^ method.

## 5. Conclusions

This study provides a comprehensive transcriptomic resolution of how the *C. quadricarinatus* hepatopancreas navigates acute MF exposure, revealing profound sexually dimorphic survival strategies. While normal females and males strictly prioritize reproductive energy allocation and pharmacological detoxification, respectively, the intersex phenotype exhibits a massive, hypersensitive transcriptomic shockwave driven by an inherent genotypic–phenotypic conflict. This extreme vulnerability is mechanistically rooted in aberrant receptor dynamics, characterized by the negative feedback collapse of the *Met*–*Kr-h1* axis and the specific remodeling of the *EcR*/*E75* pathway. To potentially alleviate severe cellular damage under such severe endocrine tension, the intersex hepatopancreas uniquely suppresses typical apoptotic clearance, relying instead on a compensatory network of de novo amino acid synthesis, sulfur relay systems, and gap junction-mediated metabolic syncytia. Furthermore, network analysis pinpointed *VCP*, *maf*, and *hdac8* as the core regulatory hubs governing proteostasis, oxidative stress, and epigenetic phenotypic lockdown. Ultimately, these findings not only explain the evolutionary persistence of intersex crayfish in volatile environments by highlighting their robust physiological buffering capacity but also identify precise molecular targets for the future development of sustainable, non-invasive monosex biotechnologies in aquaculture. Despite these robust transcriptomic insights, certain limitations of the current study must be acknowledged. First, while utilizing three biological replicates (*n* = 3) per group is the standard practice for transcriptomic profiling, it represents a minimal sample size. However, the remarkably tight intra-group clustering observed in our Principal Component Analysis (Figure S1) and the subsequent orthogonal validation via qRT-PCR (Figure S6) provide high confidence in the statistical robustness of the identified expression trends. Furthermore, the primary evidence presented herein is based on RNA-seq and co-expression network analysis, which inherently provides predictive functional trajectories rather than direct physiological endpoints. Notably, our hypotheses regarding the suppression of apoptosis and the maintenance of structural integrity in the intersex hepatopancreas under MF challenge require future morphological and protein-level validation to definitively bridge the gap between transcriptomic signaling and tissue-level pathology.

## Data Availability

The raw transcriptomic sequencing data generated in this study have been deposited in the China National GeneBank Sequence Archive (CNSA) https://db.cngb.org/cnsa/ under the project accession number CNP0009385. These data will be released and made publicly available upon the publication of this study. Further inquiries can be directed to the corresponding author.

## Supplementary Materials

The following supporting information can be downloaded at: https://www.mdpi.com/article/10.3390/ijms27094005/s1.

## Abbreviations

The following abbreviations are used in this manuscript:

AREs	Antioxidant response elements
bHLH-PAS	Basic helix-loop-helix Per-ARNT-Sim
bZIP	Basic leucine zipper
CON	Control
CYP	Cytochrome P450
DEG	Differentially expressed gene
EcR	Ecdysone receptor
EDC	Endocrine disrupting chemical
ER	Endoplasmic reticulum
ERAD	ER-associated degradation
FDR	False discovery rate
FPKM	Fragments Per Kilobase of transcript per Million mapped reads
HDAC8	Histone deacetylase 8
IAG	Insulin-like androgenic gland hormone
JHE-L	Juvenile hormone esterase-like
KEGG	Kyoto Encyclopedia of Genes and Genomes
KME	Intra-modular connectivity
Kr-h1	Krüppel homolog 1
ME	Module eigengene
Met	Methoprene-tolerant
MF	Methyl farnesoate
MFE	Methyl farnesoate esterase
PBS	Phosphate-buffered saline
RNAi	RNA interference
ROS	Reactive oxygen species
TCA	Tricarboxylic acid
UPR	Unfolded protein response
VCP	Valosin-containing protein
VgR	Vitellogenin receptor
WGCNA	Weighted Gene Co-expression Network Analysis
