# Supplementary material for: Sexually Dimorphic and Intersex-Specific Transcriptional Responses in Cherax quadricarinatus Hepatopancreas Following Methyl Farnesoate Exposure
Source: Int J Mol Sci. 2026 Apr 29;27(9):4005. doi: 10.3390/ijms27094005 (PMC13163271; doi:10.3390/ijms27094005)
Supplement: Supplementary file 1 [file ijms-27-04005-s001.zip › Supplementary Figure.pdf]

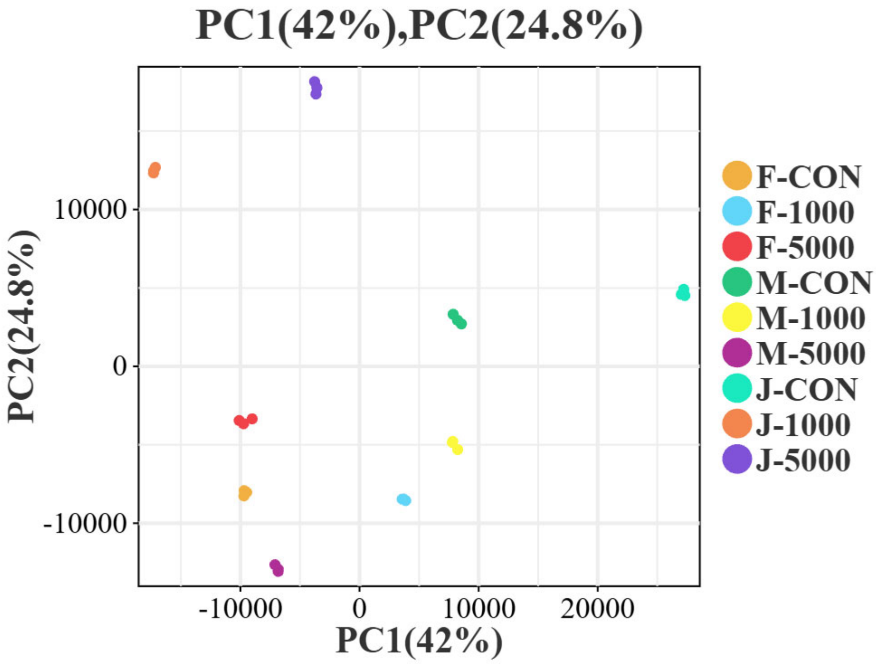

Figure S1. Principal Component Analysis (PCA) of global transcriptomic profiles across distinct sexual phenotypes and MF exposure doses.

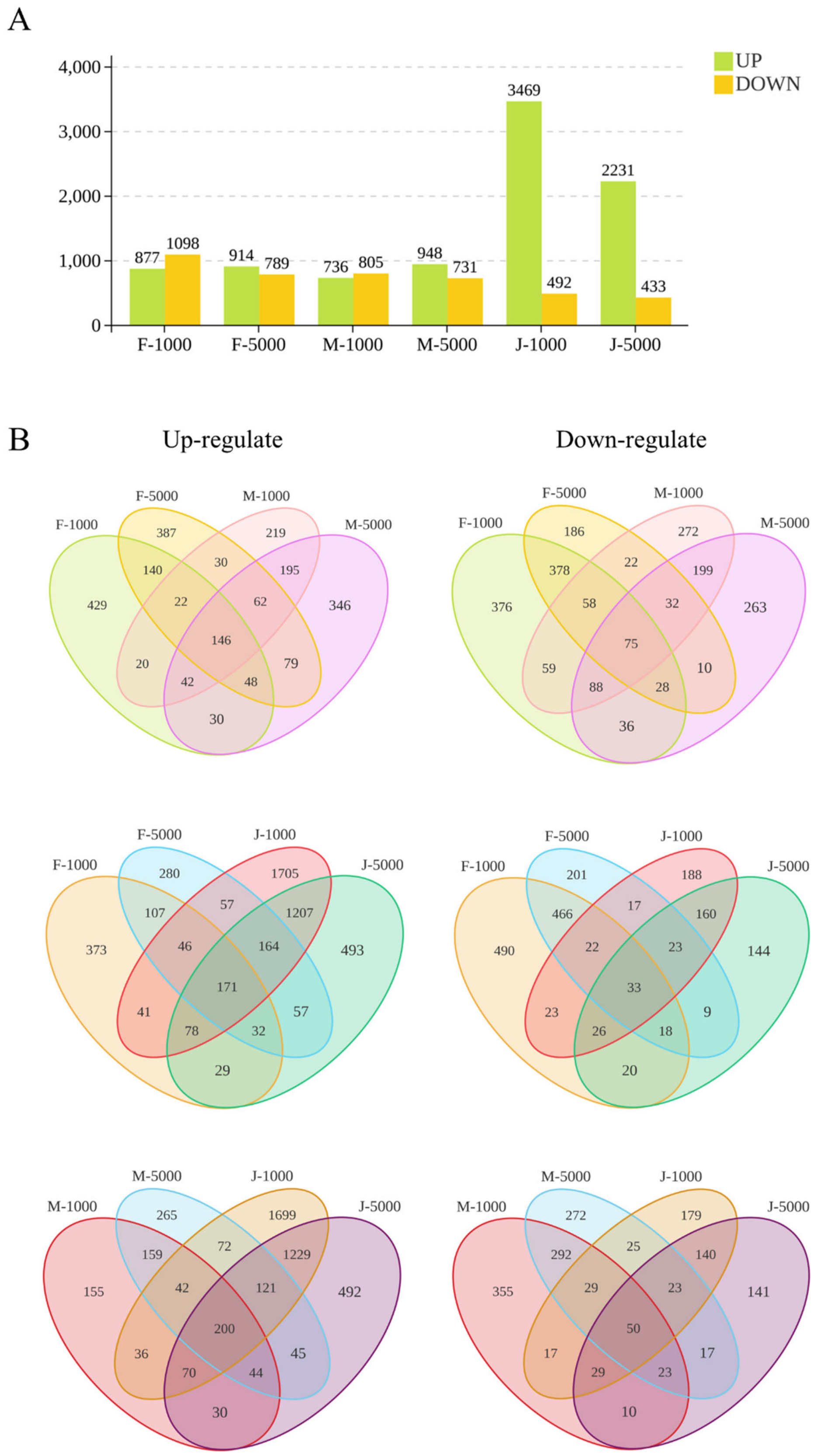

Figure S2. Statistical summary (A) and Venn diagrams (B) detailing the overlap of DEGs among different sex and MF dose combinations.

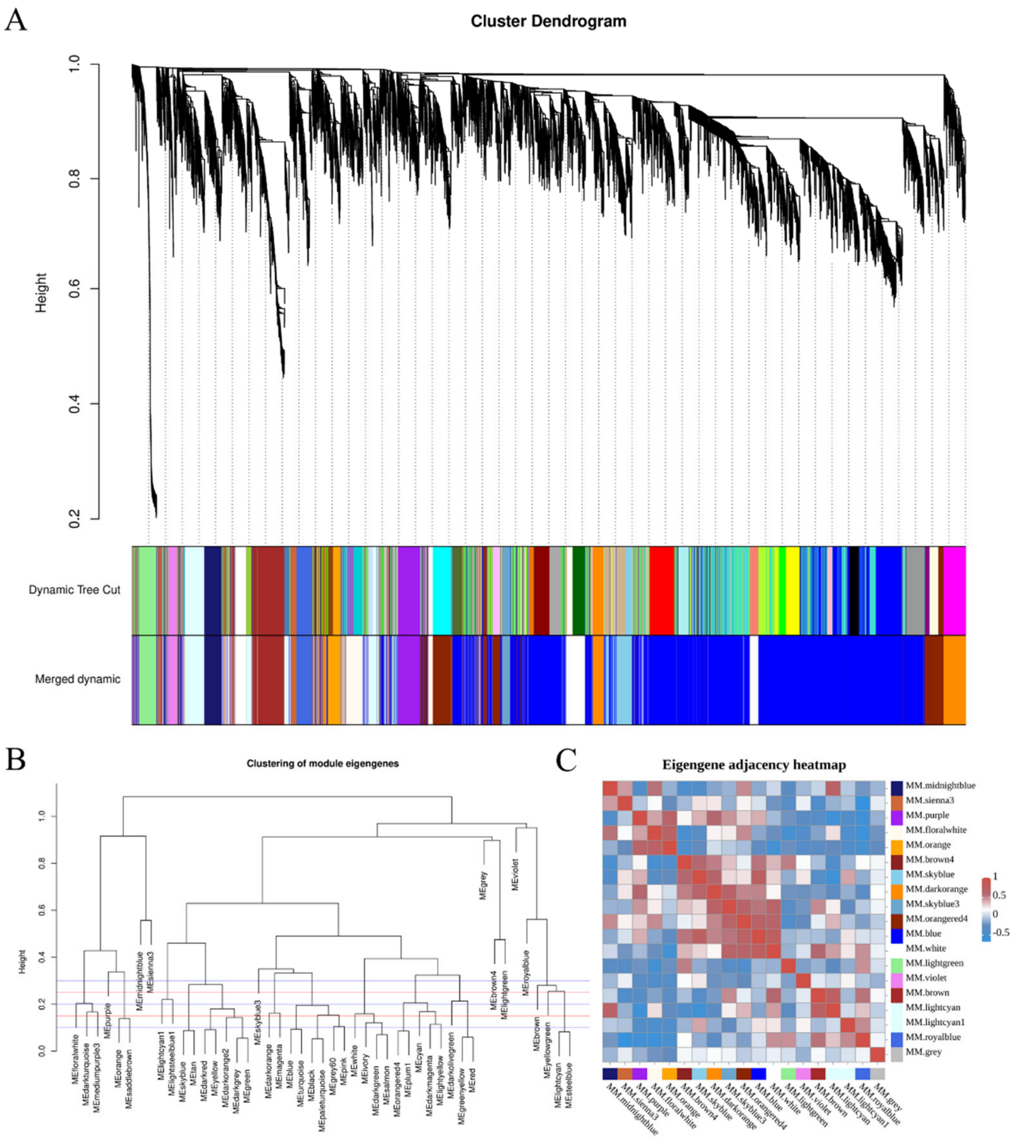

Figure S3. Construction of the Weighted Gene Co-expression Network Analysis (WGCNA) and module eigengene relationships. (A) Hierarchical cluster dendrogram of hepatopancreatic expressed genes based on topological overlap, with the assigned module colors indicated below. (B) Hierarchical clustering dendrogram of module eigengenes illustrating the relatedness among different co-expression modules. (C) Eigengene adjacency heatmap displaying the correlation matrix between the identified modules.

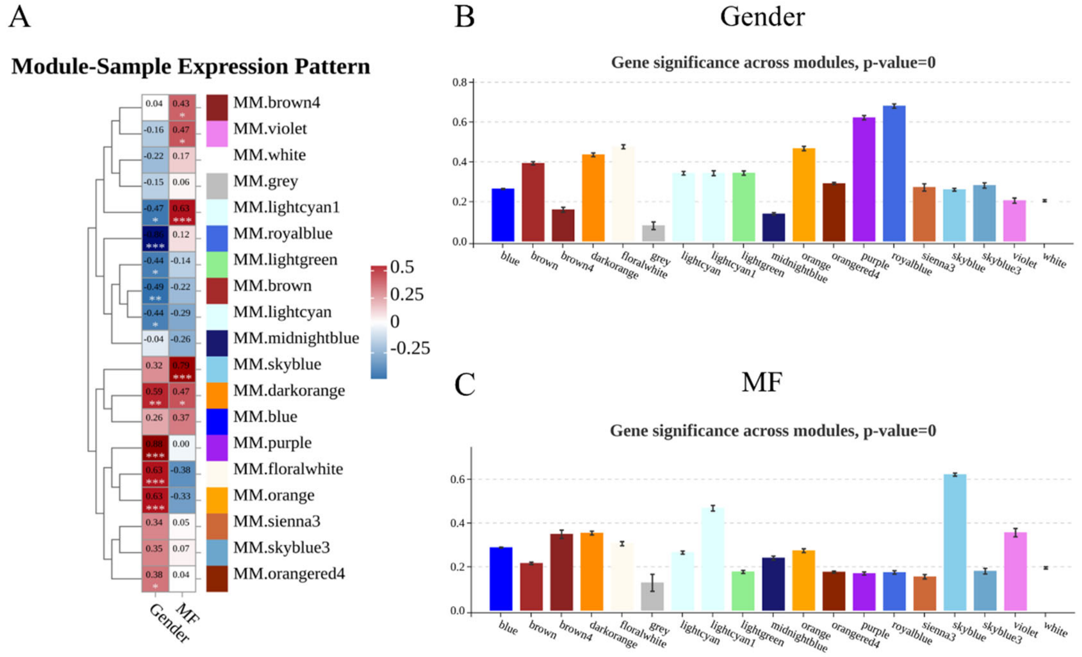

Figure S4. Module-trait relationships and gene significance analysis associated with Gender and MF exposure. (A) Heatmap of module-trait relationships illustrating the Pearson correlation coefficients between the identified co-expression modules and the specific phenotypic traits (Gender and MF dose). (B) Bar plot showing the distribution of gene significance across different modules for the trait "Gender". (C) Bar plot showing the distribution of gene significance across different modules for the trait "MF".

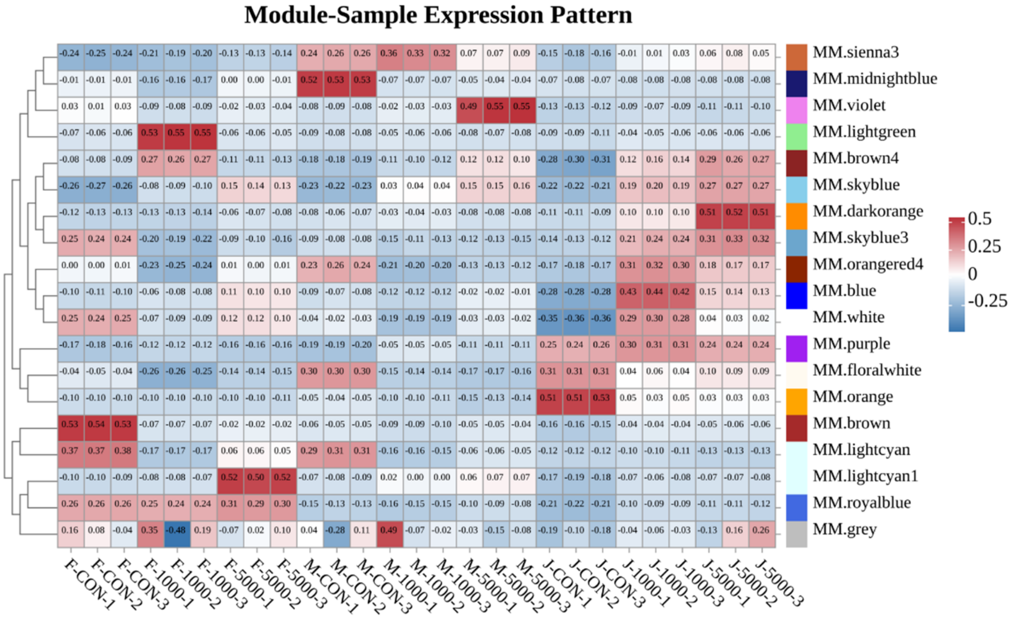

Figure S5. Module-sample expression heatmap displaying the detailed expression profiles of

identified WGCNA modules across all experimental groups.

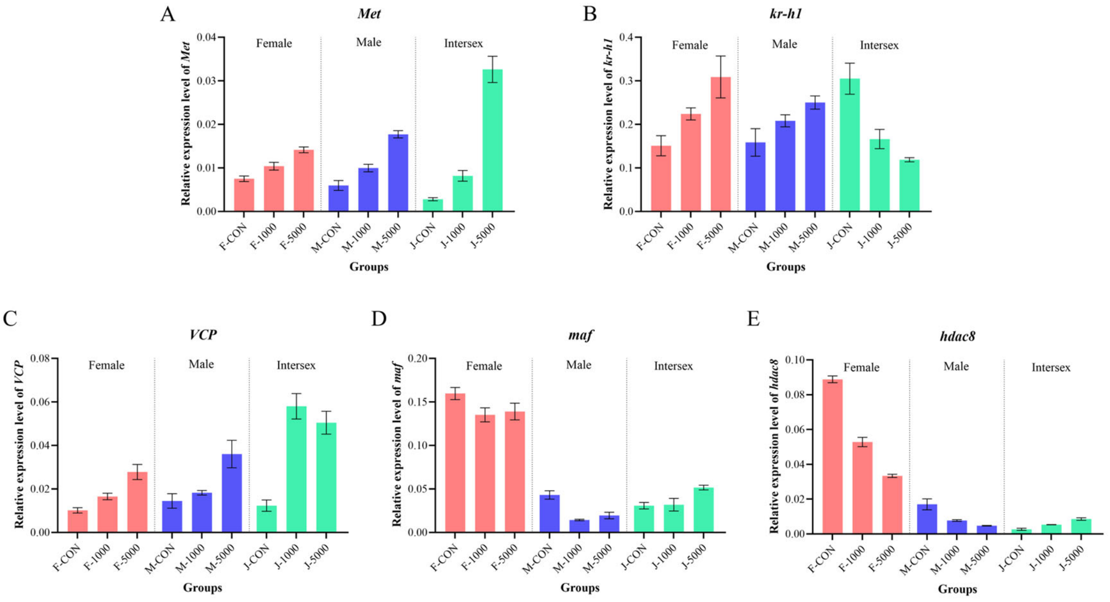

Figure S6. qRT-PCR validation of the transcriptomic profiles for the hormone receptor axis and WGCNA core hub genes (A: *Met*, B: *Kr-h1*, C: *VCP*, D: *maf*, and E: *hdac8*).
